# Counterfactual history

**DOI:** 10.1073/pnas.2519485122

**Published:** 2025-09-19

**Authors:** James Rodger Fleming

**Affiliations:** ^a^Charles A. Dana Professor of Science, Technology, and Society, Emeritus, Colby College, Centre Hall, PA 16828

History matters, and counterfactual scenarios, while entertaining to some, are just that—merely entertaining. Santer et al. (1) argue that modern atmospheric monitoring techniques, imposed retroactively on 19th-century data, could have detected climate change by 1894 “if scientists in the 19th century had the current models and observing network.” That is a big “if,” and it is counterfactual. Foote, Tyndall, and their predecessors lacked detailed measurements and understanding of radiant heat, as did Arrhenius a generation later, according to Fleming (2). Arhennius (3), citing Arvid Högbom’s 1894 model of the carbon cycle, asserted that a secular rise in CO_2_ concentration due to industrial emissions was of no concern to him. G.S. Callendar brought attention to the anthropogenic greenhouse effect in 1938 but lacked the data to convince others (4).

If the Confederacy was in possession of modern military equipment, they certainly would have prevailed in the US Civil War. If I had invested in Microsoft stock in 1988, I would be a multimillionaire today.

Scientists and historians have much to learn from one another. I encourage fruitful collaborations between them but regret the counterfactual spinning of gossamer webs found here.
